# Report from the Poor-Law Commissioners on an Enquiry into the Sanitary Condition of the Labouring Population of Great Britain

**Published:** 1843-04-01

**Authors:** 


### Report from the Poor Law Commissioners on an Inquiry into the Sanitary Condition of the Labouring Population of Great Britain. Pp. 457. London, 1842.

That the greater number of the ailments to which mankind are subject are entailed upon them by their ignorance, carelessness, and apathy, is an observation familiar to every reflecting practitioner of medicine. How large a proportion of the patients whom he daily visits, might, by the simplest hygienic precautions, have altogether avoided the maladies they are suffering under, or have rendered their duration less prolonged, and their character less grave! The public at large fares no better, and Medical Police or Public Hygiene, founded upon the deductions of science and the results of experience, and regulated by an enlightened legislation, exists not among us. And yet how pressing the necessity! It is true the metropolis—thanks to the purifying effects of the great fire—is no longer periodically decimated by the ravages of the Plague; yet is it in many of its localities, as are most of our large towns, and many rural districts, afflicted by epidemic visitations of typhus, small-pox, and malignant scarlet fever, and that sufficiently frequently, one would have thought, to have aroused not merely the exertions of philanthropic humanity to the relief of the attendant sufferings, but also to have alarmed selfish or careless apathy into the endeavour of providing preventives and precautions. When it is recollected the unprepared state the cholera found us in, it might reasonably be expected that a lesson was then read, not to be soon forgotten. More than ten years, however, have elapsed since its invasion of our shores, and we much question whether we are now, in any particular, in a better precautionary condition than we then were. It is not until the enemy has entered our camp that we become aroused to the necessity of opposing his progress; and what is done imperfectly, under the influence of a panic, might have been effected far more completely, had but a proper foresight prevailed. Much scrubbing, brushing, cleansing, whitewashing, and consumption of chloride of lime were then directed to the removal of the disgusting effects accumulated by a culpable negligence, but we have yet to learn how far the sources of these have been attacked, and their recurrence rendered impossible. And, yet, if there is one axiom more indisputable than another in medical experience, it is that where filth and dirt prevail, that where the neglected habitations of a crowded, squalid, and wretched population exist, there will be especially found the ravages of epidemic disease to prevail—so that the direction of its progress over a town or a district may be unerringly predicted by one acquainted with the topography of the misery of the neighbourhood. The experience of our own and other European countries in former times, and of the latest observers in the East, as Clot Bey and Aubert at the present day, prove this of the Plague, while the cholera is a pregnant instance of the same fact: but, while these are startling from the rapidity of the ravages they produce, the more ordinary epidemics are in reality more alarming from their permanence, (often becoming truly endemic) and from the fact of their slower operation challenging in a less marked degree the public attention.

This is, moreover, no barren fact, for the position that these physical conditions do generate or propagate epidemic disease, is scarcely less easy of demonstration than that they are for the most part *removable*. The object of the work now under review, is to point out to the public the extent of the injurious operation of these circumstances, and the most feasible plans for removing them and counteracting their influence on the well-being of society.

The Poor-Law Commissioners having had their attention directed to the great prevalence of fever (1838-9) in some of the thickly-peopled districts of the metropolis, directed Drs. Arnott, Kay, and Southwood Smith to examine into the subject, and to furnish reports how far certain physical causes of such prevalence, susceptible of removal, existed. The valuable documents so produced gave rise to an order of the House of Lords, that similar enquiries should be instituted throughout England by the Commissioners. Circulars, containing the necessary queries were accordingly addressed by them to all the medical officers, Boards of Guardians, and Assistant Commissioners of the various Unions, and in due time these produced a mass of very important, and frequently very able reports. The Marquis of Normanby subsequently desired that the investigation should also be extended to Scotland, and although the same mechanism for obtaining information did not there exist, the Commissioners received cheerful aid in their important labours from the medical profession and magistrates of the various localities. Having received the various replies, which altogether would form a most ponderous tome, and which, if indiscriminately published, would have shared the fate of various other unreadable "blue books," the Commissioners, in the practical spirit which originated the inquiry, directed their secretary, Mr. Chadwick, to prepare a digest or summary of their contents, and of the conclusions fairly deducible from these. Most ably has this talented and indefatigable officer performed his laborious task, and the result is the production of a book abounding in valuable practical information, and startling evidence of the reality of the evils, deplored, unencumbered by unnecessary details, of a convenient size, and eminently suggestive of future improvements. It is a work which ought to produce, and which has produced, a deep impression upon society, and, secure of an abundant circulation, it must prove of great utility, if anything can, in awakening the public from its lethargic condition, and in arousing it to the necessity of no longer deferring active interference.

We rejoice that the subjects of contention between the Poor-Law authorities and the medical profession are upon the eve of adjustment, and we cannot but think that the Commissioners have manifested not only much injustice, but also a great want of tact in the manner in which they have treated us. They had enough of interested prejudices, absurd misrepresentations, and the essential obstacles to all organic changes, to contend against, without arousing, by their dictatorial tone, their degrading propositions, and impossible requirements, the insulted feeling of a whole profession, which least of any can have the reproach of selfishness cast upon it, and which, by its enlightenment, and great influence with poor and rich, might have been powerfully coadjutory in working out the great social revolution the poor-law professes to achieve, or at all events assist in obtaining for it that fair-play which every great and well-intended experiment is entitled to demand. Acting harmoniously with the central authorities, what an admirable mechanism the medical officers of the poor-law unions present for ascertaining the defective condition of the public health and its causes, and for suggesting and carrying out the application of its remedies. The production of the present Report, compiled, as it is, chiefly from the gratuitous (to the injustice of this we shall again refer) reports of these officers, is a sufficient testimony of the value of such services.

To proceed to the work. We will pass its various chapters in brief review, dwelling on but few portions, as, although all that the book contains requires to be brought before the general reader, the greater portion is well known to, and would be superfluous for the professional one. We cannot do better than commence by extracting Mr. Chadwick's statement of the extent of the evil.

"A conception may be formed of the aggregate effects of the several causes of mortality from the fact, that of the deaths caused during one year in England and Wales by epidemic, endemic, and contagious diseases, including fever, typhus, and scarlatina, amounting to 56,461, the great proportion of which are proved to be preventible, it may be said that the effect is as if the whole county of Westmoreland, now containing 56,469 souls, or the whole county of Huntingdonshire, or any other equivalent district, were entirely depopulated annually, and were only occupied by the growth of a new and feeble population living under the fears of a similar visitation. The annual slaughter in England and Wales from preventible causes of typhus which attacks persons in the vigour of life, appears to be double the amount of what was suffered by the allied armies in the battle of Waterloo. It will be shown that diseases such as those which now prevail on land, did, within the experience of persons still living, formerly prevail to a greater extent at sea, and have since been prevented by sanitary regulations; and that when they did so prevail in ships of war, the deaths from them were more than double in amount of the deaths in battle. But the number of persons who die is to be taken also as the indication of the much greater number of persons who fall sick, and who, although they escape, are subjected to the suffering and loss occasioned by attacks of disease. Thus it was found, on the original inquiry in the metropolis, that the deaths from fever amounted to 1 in 10 of the number attacked. If this proportion held equally throughout the country, then a quarter of a million of persons will have been subjected to loss and suffering from an attack of fever during the year; and in so far as the proportion of attacks to deaths is diminished, so it appears from the reports is the intensity and suffering from the disease generally increased. It appears that the extremes of mortality at the Small-Pox Hospital, in London, amongst those attacked, have been 15 per cent. and 42 per cent. But if, according to other statements, the average mortality be taken at 1 in 5, or 20 per cent., the number of persons attacked in England and Wales during the year of the return, must amount to 16,000 persons killed, and more than 80,000 persons subjected to the sufferings of disease, including, in the case of the labouring classes, the loss of labour and long-continued debility; and, in respect to all classes, often permanent disfigurement, and occasionally the loss of sight." 4.

### I. GENERAL CONDITION OF THE RESIDENCES OF THE LABOURING CLASSES, WHERE DISEASE IS FOUND TO BE THE MOST PREVALENT.

The unvarying evidence of all the reports proves these to be in the most wretched condition, as regards cleanliness, ventilation, drainage, and all the circumstances necessary for even the production of the lowest degree of comfort. In numerous cases they are totally unfit for human beings to exist in. The evil is of no local or district character, but prevails wherever the poor are congregated together. So far from being peculiar to the metropolis and other large towns, it is found as prevalent at Windsor in the vicinity of the abode of royalty, as elsewhere. There typhus reigns triumphant amidst stinking ditches, undrained and overcrowded abodes, and a defective supply of water. Many apparently advantageous sites are nowise exempt. Thus Mr. Little, the surgeon of the Chippenham Union, speaking of the parish of Colerne, says:—

"From its commanding position (being situated upon a high hill) it has an appearance of health and cheerfulness which delight the eye of the traveller, who commands a view of it from the Great Western Road, but this impression is immediately removed on entering at any point of the town. The filth, the dilapidated buildings, the squalid appearance of the majority of the lower orders, have a sickening effect upon the stranger who first visits the place. During three years attendance on the poor of this district, I have never known the small-pox, scarlatina, or the typhus fever to be absent. The situation is damp and the buildings unhealthy, and the inhabitants themselves inclined to be of dirty habits. There is also a great want of drainage." 11.

Birmingham, on the contrary, where many occupations reputed unhealthy are carried on, by reason of its excellent drainage and careful building, enjoys, according to the report of Mr. Hodgson, and the physicians of the place, a remarkable immunity from fever. To Glasgow, which Mr. Chadwick visited in company with Drs. Alison, Arnott, and Cowan, seems to be extended the palm of pre-eminent filthiness, while the prevalence of typhus in that city is well known to our readers. Describing some of the wretched localities they inspected, Dr. Arnott expresses himself as follows:—

"There were no privies or drains there, and the dung-heaps received all the filth which the swarm of wretched inhabitants could give; and we learned that a considerable part of the rent of the houses was paid by the produce of the dung-heaps. Thus, worse off than wild animals, many of which withdraw to a distance and conceal their ordure, the dwellers in these courts had converted their shame into a kind of money by which their lodging was to be paid. The interiors of these houses and their inmates corresponded with their exteriors. We saw half-dressed wretches crowded together to be warmed; and in one bed, although in the middle of the day, several women were imprisoned under a blanket, because as many others who had on their backs all the articles of dress that belonged to the party were then out of doors in the streets." 24.

In connexion with this division of the subject, although the section concerning them is placed by Mr. Chadwick at the end of his work, we may briefly advert to the state of the common *lodging-houses* of the lowest classes.

"From almost every town, from whence sanitary reports have been received, that have been the results of careful examinations, the common lodging-houses are pointed out as *foci* of contagious disease within the district. These houses are stages for the various orders of tramps and mendicants who traverse the country from one end to the other, and spread physical pestilence, as well as moral depravation. The evidence everywhere received distinguishes them prominently as the subjects of immediate and decidedly strong legislative interference for the public protection." 357.

And really the horrors of these places are indescribable and their continuance disgraceful to a civilised community. In 47 of these in Birmingham, the sexes slept indiscriminately together. In one examined at Brighton, in the largest sleeping-room, 16 feet by 10, and seven high, six beds accommodated twelve persons besides children.

"It is the invariable practice," says Dr. Baron Howard, of Manchester, "for these keepers of 'fever beds,' as the proprietors were termed by Dr. Ferrier, to cram as many beds into each room as it can possibly be made to hold. The scene which these places present at night, is one of the most lamentable description: the crowded state of the beds, filled promiscuously with men, women, and children: the floor covered with the filthy and ragged clothes, they have just put off, and with their various bundles and packages, containing all the property they possess, mark the depraved and blunted state of their feelings, and the moral and social disorder which exists. The suffocating stench and heat of the atmosphere are almost intolerable to a person coming from the open air, and plainly indicate its insalubrity." "I have seen seven persons," says Mr. Rayner, of Stockport, "in the same bed, and last week removed to the infirmary a case of rheumatic fever, with translation of disease to the heart, from a bed which every night contains eight persons." "I have known," says Mr. Oliver, of Durham, "forty persons, half-clothed, lodged in one of these wretched dwellings, three or four lying in one bed upon straw, and only a single counterpane, which is never changed, to cover them."

A regular system of inspection and licensing of these places has been put in force in Glasgow recently with the best effect. "It were only a statement," says Mr. Chadwick, "of the concurrent opinions of the commissioners of police, of magistrates, of medical officers, and of the guardians charged with the administration of the poors' rates, to represent the urgent necessity of legislative provisions for the general adoption of similar measures throughout the country."

### II. Public Arrangements External to the Residences by which the Sanitary Condition of the Labouring Population is affected.

*Drainage and Sewerage.*—Unless means be secured for the removal of superfluous moisture and the refuse and filth of society it is evidently quite impossible that its healthy condition can be maintained; and yet the work now before us amply demonstrates how ineffectually this has been performed in almost every part of the country. Never were cause and effect more clearly demonstrated, for while the progress of fever in a row of houses or street has frequently been limited to those habitations, which were destitute of sewerage, and arrested at those better provided for in this respect; so, on the other hand, localities in which it had become quite endemic, have been restored to a sanitary condition when an efficient drainage, &c. has been instituted. Perhaps no town in the kingdom is effectually drained throughout, and where it is so at all, it is frequently indebted to some accident of site. The largest towns suffer most, from having such numerous crowded localities. In Liverpool, (*e. g.*) the houses of the majority of the working classes are without any sewers whatever, and from 35,000 to 40,000 of its inhabitants exist in 8,000 cellars. Out or 6,571 of these the surveyors reported 2,988 to be wet or damp, and nearly one-third of the whole are situated from five to six feet below the level of the street. Country districts, however, are by no means exempt from the same defects and their consequences, both of which have been repeatedly observed in detached residences and groups of houses.

One would have thought that where so vast an area, and so complicated a surface as the Metropolis had to be drained, and that the sewers of its now almost innumerable and continually encreasing streets required to be brought into connexion with the main ducts, that the first engineering skill which could be procured would be put into requisition for their contrivance and construction, that all the resources of science would be freely employed, and that the governance of the whole would be conducted upon general and comprehensive principles by some competent and responsible authority.

No such thing. A large portion of the capital is quite unprovided with sewers or drains of any description, the remainder is divided among a number of irresponsible and despotic bodies, termed Commissioners of Sewers, who, with a jobbing and disgracefully extravagant expenditure, employ unskilled agents, (with one exception, the Holborn and Finsbury Division,) whose proceedings, they, themselves being profoundly ignorant of all such matters, can neither direct nor controul, and the consequence is, that the operations of one commission frequently interferes with that of another, the sewers are so badly constructed as to become receptacles of filth, instead of channels for its removal, obstacles are thrown in the way of the communication of each house with the sewer; and thus, after being heavily taxed, to provide these persons with lucrative places, it frequently happens that in Winter and rainy weather whole districts are overflown by the obstruction of the sewers, while, in the hot days of Summer, the stench from these receptacles is insufferable.

"The sewerage of the Metropolis," says the author, "though it is a frequent boast to those who have not examined its operations and defects, will be found to be a vast monument of defective administration, of lavish expenditure, and extremely defective execution. In the course of the present inquiry instances have been frequently presented of fevers and deaths occasioned by the escape of gas from the sewers into the streets and houses. In the evidence given before the Committee of the House of Commons, 1834, one medical witness stated that of all cases of severe typhus that he had seen, eight-tenths were either in houses of which the drains from the sewers were untrapped, or which, being trapped, were situated opposite gully-holes: and he mentioned instances where servants sleeping in the lower rooms of houses were invariably attacked with fever." 55.

One of the difficulties in the due purification of a large capital, must always be the removal and disposal of the human excrement and other house filth. A small proportion only of the houses in London possess water-closets, and accumulations occur, therefore, in the privies and cesspools. The expense and disgusting annoyance of having these emptied cause them to become overcharged with their contents; and abundant evidence is given in this book of the horrible filthy state which frequently results. The soil often choaking up all issues, and exuding into neighbouring premises, is in the lowest tenements found dropping about and infecting the atmosphere on every side. Mr. Chadwick believes the grand agent for the removal of all impurities from the roads and houses should be abundant supplies of water, allowed to flow with some degree of impulse, and no one can doubt the preferability of washing, in this manner, such impurities away under-ground, to conveying them by hand-labour, and exposing a whole locality to all the horrors of these night operations. But there is some difficulty in ultimately disposing of them, for if washed into the Thames, of course they become additional sources of pollution to that already dirty river. But we think its water is already and unavoidably unfitted for human consumption, unless it be previously purified, or the quantity required drawn from a point sufficiently removed from the seats of impurity, and, therefore, that there can exist no valid objection to conveying into its bosom the immense accumulations resulting from the refuse of a population of near two millions: and, indeed, we ought to feel rejoiced at having so magnificent a main sewer at hand for the purpose. Mr. Chadwick, however, objects to this proceeding, on the ground that so much good stuff should not be wasted; and he suggests that it should be conveyed by pipes to reservoirs, for the purpose of employing it as manure in some of the barren rural districts; furnishing, as it does, when diluted with water, an excellent material for irrigation, as is found by experience, in the vicinity of Edinburgh—and, we believe we may add, in the Celestial Empire. He says—

"The condition of large rural districts in the immediate vicinity of the towns, and of the poorest districts of the towns themselves, presents a singular contrast in the nature of the agencies by which the health of the inhabitants is impaired. Within the towns we find the houses and streets filthy, the air fetid, disease, typhus, and other epidemics rife among the population, bringing, in their train destitution, and the need of pecuniary as well as medical relief; all mainly arising from the presence of the richest materials of production, the complete absence of which would, in a great measure, restore health, avert the recurrence of disease, and, if properly applied, would promote abundance, cheapen food, and increase the demand for beneficial labour. Outside the afflicted districts, and at a short distance from them, as in the adjacent rural districts, we find the aspect of the country poor, and thinly clad with vegetation, except rushes and plants favoured by a superabundance of moisture, the crops meagre, the labouring agricultural population few, and afflicted with rheumatism and other maladies, arising from damp and an excess of water, which, if removed, would relieve them from a cause of disease, the land from an impediment to production, and, if conveyed for the use of the town population, would give that population the element of which they stand in peculiar need, as a means to relieve them from that which is their own cause of depression, and return it for use on the land as a means of the highest fertility." 97.

*Supply of Water.*—It is impossible that cleanliness and health can be secured unless this is abundant, for when it is scanty, dirty habits become necessarily engendered, and not a few maladies originate in personal dirtiness. There is, perhaps, hardly a town in the kingdom but has reason to complain on this score. "No previous investigations," says Mr. Chadwick, "had led me to conceive the great extent to which the labouring classes are subjected to privations, not only of water for the purpose of ablution, house cleaning, and sewerage, but of wholesome water for drinking, and culinary purposes."

The mere circumstance of water existing in a well or a river, even in the nearest vicinity, is not sufficient. To be effectually useful it must be brought into the house in pipes; and, certainly, this is even now done at a very cheap rate in London, for Mr. Chadwick calculates 136 pail-fuls of water may be obtained for five farthings, and the same quantity of water is delivered by the London Companies for sixpence as by the Paris water carriers for nine shillings. Cheap as this is, it is yet beyond the means of many of the lowest classes, and means of effecting the supply more economically and more universally, as a matter of public health, rather than of commercial profit, have yet to be discovered. Private companies cannot afford this at a lower rate than they do, and they will not extend and force their commodity into localities which are not likely to prove remunerative. We are accustomed to prefer the competition of private companies in our country to the operations of government in continental states, and generally with reason, but it will often be found that the wealthy and middle classes are far more benefited by this state of things than those of the lower orders, who can offer no remunerative return.

### III. Circumstances in the Internal Economy and Bad Ventilation of Places of Work: Workmen's Lodging Houses, Dwellings, and the Domestic Habits affecting the Health of the Labouring Classes.

*Overcrowding and Defective Ventilation.*—As a strong example of the ill effects of these, Mr. Chadwick brings forward the case of the metropolitan tailors, his attention having been especially directed to them by reason of the great preponderance of early deaths, and cases of widowhood and orphanage. The inquiry he instituted was indeed called for. Some of the great London tailors employ from 80 to 250 men, who are congregated together in crowded apartments, so that each man is almost in contact with his neighbour. What with the odour of the profuse perspiration which is always present, and the more perceptible from the half undressed state in which these men work, the great heat of the room, and the utter absence of ventilation, such an atmosphere is produced that new comers frequently faint away, while the old hands are in the habit of stimulating their exhausted strength by repeated libations of gin and beer during the day, which are regularly brought to them, engendering thereby all the horrors of intemperance. In such a state is the surface of the bodies of these poor men that the employers sometimes have £40. or £50. worth of light goods spoiled in a season by the perspiration alone. The witnesses considered that two out of twelve hours work were lost by bad work from nervous exhaustion, which, in a well-ventilated shop need not be present. A man, coming into one of these shops at 20 is a worse man at 40 than he would be, if working at a country tailor's at 50, and indeed not above 10 in the 100 are to be found in the London shops above 50. All this results from the ignorance, and not from the want of generosity on the part of the employers, who have contributed munificently, for decayed tailors, sums, indeed, far less than which, if expended in sanitary contrivances, would have prevented the premature arrival of a diseased old age, and would have enabled the tailor to have provided means for his own subsistence against the advent of the natural period of his decay.

By examination of the registries it is found that 53 per cent. of these operatives die of disease of the respiratory organs in the metropolis, and but 39 per cent. in the country, 5 per cent. of typhus in London, and 1 per cent. in the country, and that while in town only 12 in 100 (and these including master-tailors) attain old age, in the country 25 in the 100 do so. Another class, much oppressed by these causes of disease, in the metropolis, is that of the milliners and dress-makers, and the more so as much of their work, in large shops, is performed at night, when the air is additionally deteriorated by the gas or candles.

Medical readers require no arguments to prove the baneful and destructive effects of over-crowding and ill-ventilation. The experience of the whole profession, as ascertained through the medium of the army, of hospitals, foundling-hospitals, and even of private practice, is unanimous on this point. The second Report of the Poor-Law Commissioners contained a recent case. A large pauper school attracted great attention by reason of the great mortality prevailing in it, and which was at once attributed to defective nourishment. On a searching inquiry, however, being instituted, the diet was found to be unusually good, but due ventilation wholly unprovided for. This was remedied and "in the same space where 700 children were by illness awakening extensive sympathy, 1100 now enjoy excellent health."

One effect of diffusing information upon this subject will be to disabuse the public mind as to the insalubrious effects of certain occupations *per se*, proving such effects usually to result from the prejudicial but non-essential circumstances under which they are usually carried on. The inspectors are enabled to distinguish the workers in cotton factories in country districts at once from those employed in towns. Du Chatelet exposes the error of the general opinion as to the injurious nature of the tobacco manufacture, and Mr. Chadwick confirms the truth of his remarks.

"If I might add my testimony on this point, derived from my own observations on two of the commissions of inquiry, on which I have had the honour to serve, it would be entirely in corroboration of the above statement. On comparing the actual condition of workmen with the medical description of these diseases, and the causes, we commonly found that the results of a cluster of causes are commonly ascribed to one; and in respect to several classes of workmen the real cause, the invariable antecedent, such as defective ventilation, is unnoticed. No persons were frequently more surprised than the intelligent workmen, by the frequent exaggerated accounts of the operations of particular causes upon them, and the erroneous association of effects to causes with which they were known to have no real connexion." 116.

As our business is rather with the health than with the morals of the community, we may pass over Mr. Chadwick's illustrations of the moral degradation which results from the crowded condition of the habitations of the poor in town and in country—whole families cooped up in one miserable room, and herding together, heedless of *sex or age*, like so many swine, and exposed to the most revolting violations of all decency and decorum. The vitiated air of such abodes, by its depressing effect upon the nervous system becomes powerfully inductive of the habits of dram drinking, which are so freely indulged in by both sexes. The mere wretchedness of an abode, even independently of its overcrowded state, has the tendency to reduce its inhabitants to the same level, and instances are here given of persons of exemplary conduct completely degenerating, after being placed in abodes which it was physically impossible to keep clean and comfortable, and of others who, removed from the degrading hovels in which they had vegetated, to habitations fit for human beings, underwent a complete revolution in manners and habits. And it is in this direction that the helping hand to the poor man should be first directed, for even when possessed of the pecuniary means of extricating himself, unable to appreciate comfort he has never enjoyed, he prefers doggedly to continue in his abasement to expending a trifle for his own emancipation. Thus, it is by no means an indication of a man's being in the receipt of low wages that his abode is wretched, for some of the worst provided for families, cited in this work, were well paid, while some of those possessing the neatest abodes were in the receipt of sums apparently utterly insufficient. No marked improvement of the condition of the poorer classes ever resulted from their own unaided exertions, for a certain extent of education and refinement is necessary to appreciate the importance of making some sacrifice for the obtaining this description of amendment; and, charity bestowed upon them, while they continue in their semi-brutalized condition, is frequently converted into the means of its perpetuation. The following passage is important.

"The more closely the investigation as to the causes of epidemic disease is carried the more have the grounds been narrowed on which any presumption can be raised that it is generally caused by extreme indigence, or that it could be made generally to disappear simply by grants of money. In the great mass of cases in every part of the country, in the rural districts, and in the places of commercial pressure, the attacks of disease are upon those in full employment, the attack of fever precedes the destitution, not the destitution the disease. There is strong evidence of the existence of a large class of persons in severe penury in some places, as in Glasgow, being subject to fever, but the fever patients did not, as a class, present evidence of being in destitution in any of the places we examined." 144.

The evidence of Mr. Byles, (as also of the majority of the reporters) medical officer of Whitechapel Union, concerning the Spitalfields district, is corroborative of the above. He found fever chiefly prevalent among those shut up in full employment, and not among those destitute, and roaming about in quest of it. As observed above, Mr. Chadwick, looking upon fever not usually as a consequence of destitution, but as leading to it, believes that the prevalence of a contrary opinion has prevented vigorous means being put into operation for the removal of those causes of the disease which are susceptible of remedies.

### IV. Comparative Chances of Life in different Classes of the Community.

"Very dangerous errors arise from statistical returns and insurance tables of the mean chances of life made up from gross returns of the mortality prevalent amongst large classes, who differ widely in their circumstances. Thus we find, on enquiry into the sanitary condition of the population of different districts, that the average chances of life of the people of one class in one street will be 15 years, and of another class in a street immediately adjacent, 60 years. In one district of the same town I find, on the examination of the registries, the mortality only 1 out of every 57 of the population; and in another district one out of every 28 dies annually. A return of the average or the mean of the chances of life, or the proportions of death in either instance, would and does lead to very dangerous errors, and amongst others to serious misapprehensions as to the condition of the inferior districts, and to false inferences as to the proper rates of insurance." 153.

In order to draw more accurate deductions than could be obtained under ordinary circumstances, Mr. Chadwick obtained returns from several superintendent registrars of the average mortality as it occurs in three different classes of society, and we regret that we cannot extract the whole of these. The three classes consisted of 1st, gentlemen, professional persons and their families; 2nd, tradesmen (farmers in rural districts) and families; 3rd, mechanics, servants, labourers and families. The average age of the deceased in each of these was, in the Bolton Union, 34; 23; 18: Bethnal Green, 45; 26; 16: Leeds, 44; 27; 19: Liverpool, 35; 22; 15: Whitechapel, 45; 27; 22: Strand, 43; 23; 24. Of all districts examined, the county of Rutland (the moral and intellectual condition of whose inhabitants is also favorably noticed) stands highest, 52; 41; 38. While Manchester is thus represented, 38; 20; 17. The agricultural population of Wilts is also as follows, 50; 48; 33, and in the Kendal Union, where the population is partly agricultural, partly manufacturing, the numbers are 45; 39; 34.

The difference in the proportion of deaths among all classes between one district and another shews the influence of locality markedly. Mr. Finlaison has drawn up a table to exhibit this in the respective metropolitan districts—although the extremes are more remarkable when smaller localities are contrasted.

Out of 10,000 inhabitants there were of deaths—
224 in the Western—231 in the Northern.
255 in the Central—260 in the Eastern.
259 in the Southern—247 for the whole metropolis.

The researches of M. Villermé are also quoted, who, assuming the exemption from taxation as a test of poverty, found the mortality in the various arrondissemens of Paris to be high or low according as these contained many or few exempted houses. The cholera followed the same track. Similar results attended the comparison of the mortality found in different streets accordingly as they were inhabited by different classes, and from comparing it among persons possessing similar incomes, but residing in favorable or unfavorable localities.

"If we could ascertain the rates of mortality formerly prevalent in the separate districts of each large town, it is probable that we should find that the improvement in the average chances of life of the whole town has been raised principally by the improved chances in the districts where the streets have been widened, paved, and cleansed, and the houses enlarged and drained: and that the amount of sickness and chances of life in the inferior districts are as little altered as their general physical condition. The present condition of those parts of London where the average mortality is 1 in 28 annually, appears to be not dissimilar to the general condition of the whole metropolis about a century ago, which was said to be 1 in 20, a rate still to be found in some of the most neglected streets." 172.

In Paris the same proportionate decrease in the ratio of mortality and improvement of structural arrangements is observable. M. Villermé's tables show the mortality to be 1 in 52 in one district, and 1 in 26 in another. The ratio varies in London from 1 in 28 to 1 in 57, and in the Township of Manchester (with a population of near 80,000) one twenty-eighth part of the inhabitants are annually swept away, while in a favoured suburban district only a sixty-third part perish.

Nor have we the consolation of knowing that this mortality is attended by a thinning of an excessive population, and a greater degree of prosperity for the survivors.

"If in the most crowded districts, the inference is found to be erroneous, that the extent of sickness and mortality is indicative of the pressure of population on the means of subsistence, so is the inference that the ravages act to the extent supposed, as a positive check to the increase of the numbers of the population. In such districts the fact is observable, that where the mortality is the highest, the number of births are more than sufficient to replace the deaths, however numerous they may be. * * * * We have seen that in the lowest districts of Manchester of 1000 children born, more than 570 will have died before they attain the fifth year of their age. In the lowest districts of Leeds the infant mortality is similar. This proportion of mortality M. Mallet designates as the case of a population but little advanced in civilization, ravaged by epidemics—a population in which the 'influences on the lower ages are murderous, but where the great mortality in infancy is compensated by a high degree of fecundity. It is the case of the population in many large towns, especially in past ages.' But whilst in Manchester, where $\frac{1}{28}$ of the whole population is annually swept away, the births registered amount to 1 in 26 of the population; in the county of Rutland, where the proportion of deaths is 1 in 52 of the population, the proportion of births, is only 1 to 33." 177-9.

The increase of births after a pestilence, has long been observed, and M. Quetelet's reseaches in various countries confirm the fact of an increase of births coinciding with the high mortality of the worst districts. Mr. Chadwick has made further interesting observations upon the subject.

"By means of the last census, and the last year's completed registration of deaths and births in England, I am enabled to show that there has been an increase of the population from births alone in those parts of the country where the proportionate mortality is greatest. * * *

* * * We here find * that in the 14 counties where proportionate mortality has been the *least*, the 184 deaths in 10,000 persons are made up by 297 births: hence 113, or more than one per cent., is added by new births to the existing population. In the 14 *intermediate* counties where the deaths on every 10,000 persons increase to 208, there the deaths are again made up 302 births, and 94, or close upon one per cent., are again added to the population. In the fourteen counties where the increase of the mortality is *greatest*, the deaths in each 10,000 are increased to 259; but here, also, we find that the births are again sufficient to make up for the deaths; they are 348, and increase the population by 89, or less than one per cent. Hence, if the number of births in each 10,000 persons of the fourteen counties where the mortality has been the greatest, had taken place among every 10,000 persons of the counties where the mortality has been the least, then the increase of population in these latter by births, instead of being 113, would have been 164.

"I must again observe, that the registration of births in the most populous town districts, where the mortality is greatest, is the least perfect. The excess of births over deaths may really be taken to be greater than shown in the returns from the districts where the mortality is greatest." 183.

Mr. Chadwick warns us not to estimate the strength of a people by its mere numerical condition.

"Simple enumerations of the numbers of a population are of themselves but imperfect means for judging of its progression in strength. That is best shown in the increased proportions of the adults, who are of the age and strength and skill for productive industry, in the extended period during which each adult labourer occupies his post.

"M. Mallet bears testimony that the experience of Geneva is confirmatory of the important rule, that the strength of a people does not depend on the absolute number of its population, but on the relative number of those who are of the age and strength for labour. It is proved that the real and productive value of the population has there increased in a much greater ratio than the increase in its absolute numbers. The absolute number has only doubled, in the instance of Geneva, during three centuries; but the value of the population has more than doubled upon the purely numerical increase of the population. In other words, a population of 27,000, in which the probability of life is 40 years for each individual, is more than twice as strong, for the purposes of production, as a population of 27,000 in which the probability or value of life is only twenty years for each individual.

"The important general fact of the proportion of adult physical strength to the increased duration of life, or improved sanitary condition of the individuals, is verified by the examinations of the individuals of different classes. M. Villermé states, that the difference of strength between classes such as those in which we have seen that the value of life differs, is well known to the officers engaged in recruiting for the army, but no one had collected the facts to determine the precise difference. The time allowed to M. V. only enabled him to do so at Amiens. The result was, that the men of from 20 to 21 years of age were found the more frequently unfit for the trade of arms, from their stature, constitution, and health, as they belonged to the poorer classes of manufacturing labourers. In order to obtain 100 men fit for service, it was necessary to have

---

* We omit the tables, and give only the results.

as many as 343 of the poorer classes: whilst 193 conscripts sufficed of the classes in better circumstances. Analogous facts were observed in the greater part of the towns in France in which he conducted his official investigations.

"In the evidence of recruiting officers, collected under the Factory Commission of Inquiry, it was shewn that fewer recruits of the proper strength and stature for military service are obtainable now than heretofore from Manchester. I have been informed that of those labourers now employed in the most important manufactories, whether natives or migrants to that town, the sons who are employed at the same work are generally inferior in stature to their parents. Sir James M'Grigor stated to me the fact that 'A corps levied from the agricultural districts in Wales, or the northern counties of England, will last longer than one recruited from the manufacturing towns of Birmingham and Manchester, or from near the Metropolis.' Indeed, so great and permanent is the deterioration, that out of 613 men enlisted, almost all of whom came from Birmingham, and five other neighbouring towns, only 238 were approved for service. The chances of life of the labouring classes of Spitalfields are amongst the lowest that I have met with, and there it is observed of the weavers, though not originally a large race, that they have become still more diminutive under the noxious influences to which they are subject.

"This depressing effect of adverse sanitary circumstances on the labouring strength of the population, and on its duration, is to be viewed with the greatest concern, as it is a depressing effect on that which most distinguishes the British people, and which it were a truism to say constitutes the chief strength of the nation—the bodily strength of the individuals of the labouring class. The greater portion of the wealth of the nation is derived from the labour obtained by the application of this strength, and it is only those who have had the means, practically of comparing it with that of the population of other countries, who are aware how far the labouring population of this country is naturally distinguished above others. There is much practical evidence to show that this is not a mere illusion of national vanity, and in proof of this, I might adduce the testimony of some of the most eminent of the employers of large numbers of labourers, whose conclusions are founded on experience in directing the work of labourers from the chief countries of Europe." 186.

### V. PECUNIARY BURDENS CREATED BY THE NEGLECT OF SANITARY MEASURES.

We regret that we cannot follow Mr. Chadwick into his statistical exposition of the encreased charges upon society, in the way of the rapid multiplication of widowhood and orphanage, incidental to the premature death of working men.*

When masses of persons are exposed to noxious influences, there is found to result a young and feeble, in the place of a mature and robust population. According to the statement of Mr. Lanyon, at the Poly-

* "That of the 43,400 cases of widowhood, and 112,000 cases of destitute orphanage relieved from the poors' rates in England and Wales alone, it appears that the greatest proportion of deaths of the heads of families occurred from the above specified and other removable causes; that their ages were under 45 years; that is to say, 13 years below the natural probabilities of life, as shown by the experience of the whole population of Sweden." Recapitulation, 369.

technic Society of Cornwall, " on an examination of 2,145 men, engaged in mining, it was found that their average age was 30 years; and that the average period they had been engaged in work was 15 years. On examining the condition of 1,033 artisans, and agricultural labourers, living and working in the vicinity, it was found that their average age was 40 years, and that their average period of work, then completed, was 25 years. Of the mining population one-third only had reached 50 years of age, whilst of the non-mining population, one-third had attained 70 years of age."

Dr. Alison found that the average of the colliers of Tranent was 34, of the surrounding agricultural population 51. When the population of the lower orders of Manchester or the Metropolis are brought together in great numbers, (as in the Chartist mobs,) the preponderating numbers of youthful persons (ranging from 16 to 25,) is remarkable, and the absence of a proportion of those of middle-age still more so. These masses of juveniles form the active force in the disturbances, (as in the Bristol riots,) and strikes, bearing down by their preponderating numbers the opposition of the small minority of mature and reflecting operatives.

" The experience of the metropolitan police is also similar as to the comparatively small proportion of force available for public service from such depressed districts. It is corroborative also of the evidence as to the physical deterioration of their population, as well as the disproportion in respect to age. Two out of every three of the candidates for admission to the police force itself, are found defective in the physical qualifications. It is rare that any one of the candidates from Spitalfields, Whitechapel, or the districts where the mean duration of life is low, is found to present the requisite physical qualifications for the force, which is chiefly recruited from the open districts at the outskirts of the town, or from Norfolk and Suffolk, and other agricultural counties.

" In general the juvenile delinquents, who come from the inferior districts of the towns, are conspicuously under size. In a recent examination of juvenile delinquents at Parkhurst, by Mr. Kay Shuttleworth, the great majority were found to be deficient in physical organization. An impression is often prevalent that the criminal population consists of persons of the greatest physical strength. Instances of criminals of great strength do certainly occur; but, speaking from observation of the adult prisoners from the towns, and the convicts in the hulks, they are generally below the average standard of height." 202.

We can only extract a portion of the author's illustration of the effects of premature deaths on the number of the children, and in depressing the survivors.

" In works where the average period of working ability is extended to the natural period of superannuation, which the evidence shows that a combination of internal and external sanitary measures may be expected to give, viz. an average of full sixty years, the account for one place would be one superannuated workman and one widow, and a family of four or five well-grown children, who having received parental care during that period, will probably all have obtained, before its termination, the means of independent existence. Whereas, with a population of only fifteen or twenty years of working ability, the same place of work may, during the same period, have been filled by two generations, and one-fourth of work-people, not one of which has brought all the children dependent upon it to maturity or a condition of self-support; and the account of widowhood and orphanage will frequently, for the same place of work, stand thus:—

| Workmen prematurely dead. | | Orphan Children. | Years loss of Support. |
|---|---|---|---|
| J. M. | 1 widow | 2 | 39 |
| S. H. | 1 widow | 7 | 26 |
| H. Y. | 1 widow | 5 | 15 |

"That is to say: three widows instead of one, and three sets of stunted and unhealthy children, dependent, for such various periods, as those above specified, and competing for employment at the same place; instead of one set of healthy children, arrived at the age of working ability for self-support. The occupation of the places of work by a comparatively young and procreative population, brought forward by the premature removal of the middle-aged and the aged workers, by the various causes of premature deaths, will, I apprehend, sufficiently clearly account for the generally increased proportion of births in those districts where the rate of mortality is high; and it will scarcely be necessary to give further illustrations of the dreadful fallacy which tends to an acquiescence in the continuance of the causes of pestilence and premature mortality as corrections of the pressure of population." 205.

The mere pecuniary cost of disease is thus stated by the late Dr. Cowan.

"If any arguments were wanting to arouse the community to the investigation of this important subject, they might be drawn from the heavy pecuniary tax which fever entails on the benevolent of our city, from the poverty, crime, and misery which this disease engenders. It is not possible, from the data before me, to give anything like an accurate calculation of the sums spent in Glasgow for the treatment of fever during the last twenty years.

"The following calculation intentionally falls considerably under the amount, to prevent every suspicion of exaggeration:—

| | £. |
|---|---|
| "1. Cost of the Fever Hospital | 8,566 |
| 2. Temporary Hospitals | 5,000 |
| 3. 21,691 Patients at £1. 10*s*. treated at the expense of the Infirmary | 32,536 |
| | £. 46,102 |

"To this amount fall to be added the expense of treating the poor in their own houses, under the district surgeons of the burgh, and any sums expended by the heritors, or the gorbals and barony parishes for similar purposes. But this sum must have been greatly increased by the demands of pauperism produced by fever on our poors' rates, and on the private benevolence of our citizens; for the duration of the disease, and the period of convalescence, which must elapse before an individual can resume his work, will average rather more than six weeks, and when to this is added again the difficulty of again finding immediate employment, we may safely assume that the 12,895 treated in the fever hospitals, during the last seven years, all, with few exceptions, depending on their daily labour, and extending the benefit of that labour to others, were out of employment for a period of at least six weeks." 207.

### VI. Evidence of the Effects of Preventive Measures in Raising the Standard of Health and the Chances of Life.

Mr. Chadwick properly adduces the improved health in prisons, as compared with former times, as a good proof how far corrective means of a sanitary character may be satisfactorily attempted. He also shows that the conditions of the prisons, at the period when Howard visited them, was in no-wise worse, nor called louder for amendment, than some of the crowded neighbourhoods of the present day. It is a subject of frequent observation, that while these are sometimes ravaged at the present day by deadly epidemics, the health of the inmates of neighbouring prisons continues satisfactory. Mr. Finlaison states that, were any benefit society to employ a scale of premiums founded on prison experience, it would be insolvent in three years.

No less triumphant is the testimony afforded by the sanitary condition of the navy, for while in 1726, Admiral Hosier in sailing to the West Indies, buried his ship's company twice, and Dr. Wilson, in his Preface to the Medical Reports, observes that within the limits of the South American Command, the Centurion lost, a century ago, 200 out of 400 men from scurvy: from 1830 to 1836, the British squadron, employed in South America, lost by disease of every description only 115 out of 17,254 men. In 1779 the proportion dying of the employed was 1 in 8; in 1811, 1 in 32; from 1830 to 1836, 1 in 72.

The author enters into detail to prove that the expense, even if defrayed by the labouring classes themselves, of effecting an improved condition of their sanitary state, would be infinitely less than is entailed upon them now by loss of work incident to sickness, as shewn by their insurances made in benefit societies. And even supposing some additional demand were made upon them, its defrayal might be accomplished by diminishing their enormous expenditure for ardent spirits, tobacco, beer, &c.—amounting to about 40 or 50 million per annum.

He deprecates strongly the policy of exempting low-priced tenements from contributing to the taxes, thereby encouraging the creation of masses of wretched and ill-conditioned abodes, whose rents are only higher in consequence of such exemption. But, as was before observed, the initiative must at least be undertaken for these classes, for, what with the necessity they are under of residing in the immediate vicinity of their employment, and their inability themselves to undertake the amendments of sewerage, ventilation, and construction of their dwellings, it is obvious that they are themselves unable to *originate* (however much they may be afterwards able to confirm and profit by them) any marked improvements in these respects. The influence and power of the large employers of the manufacturing and other classes are great, and their responsibility proportionate, and we do not hesitate to say that the majority are culpably indifferent, and disgracefully negligent in providing for the mere physical comforts of those who have no other protectors. The few who have erected commodious residences for their work-people have reaped their reward, not only the richest and most gratifying of all, in beholding the vast consequent moral and physical improvement often in cases apparently hopeless, but also a reward, not to be passed over, in their improved capabilities for, and regularity at work. So that several who have commenced their improvements from purely philanthropic motives, have been surprised to find the pecuniary advantages proceed *pari passu*. One employer states that, for £7000. he would not exchange his collection of improved work-people, which he had accumulated around him in comfortable abodes, for the promiscuous assemblage usually found in similar manufactures. The payment of the operatives, too, in the place of business instead of at the public house, and at an early period of the day, has been found powerfully instrumental in promoting the happiness of themselves and families by diminishing the temptation to dissipation and extravagant expenditure—other employers have eminently contributed to the health and cleanliness of their work-people by employing some of their large supplies of water as baths, and insisting upon a certain degree of decorum and personal neatness. Again, how many means may be put into force for partially obviating the inconveniences of many employments, as of plumbers and painters, those which create dust or noxious fumes, &c.: but it must be acknowledged, that the carelessness and prejudices of the operatives themselves much impede the general adoption of these. The author likewise dwells forcibly upon the necessity and advantages of maintaining and throwing open wide spaces for public resort and amusement.

Some examples are given, also, of the erection of an improved class of cottages in the rural districts, and the same invariable good effects have followed the proceeding.

### VII. RECOGNIZED PRINCIPLES OF LEGISLATION AND STATE OF THE EXISTING LAWS FOR THE PROTECTION OF THE PUBLIC HEALTH.

The author passes in review the state of the existing laws in this country contributing to the protection of the public health, but it requires no exposure of this kind to prove to our readers that the same imperfect, piecemeal, and obsolete legislation which disgraces our country in so many other particulars, is pre-eminent in all matters relating to public hygiene. Who could have believed, until a disagreeable experience had enlightened them, that in the greatest, the most wealthy, and the most crowded metropolis of the world we should find a building-act prevail which only extended to a limited district, so that beyond this, the slight protection which party-walls give against the extension of fire is not insisted upon, and that even within the limit of its operation it is constantly evaded, and extensive conflagrations, involving great loss of life and property, continually result—that with an abundance of water, a deficiency is often found at fires, no provision for public baths exist, no employment of it for the purpose of street cleansing and purification occurs, and thousands of the poorest houses, where, on the score of health, it is most wanted, are destitute of it, though it may be rushing by their very doors in its subterraneous conduits—that any builder, who choses to indulge his caprice or his cupidity, may construct this or that alley or cul-de-sac, excluding its inmates from all view and enjoyment of exhilirating sunshine and ventilating breezes, and not even providing the dens he has created with the means of dismissing the refuse of the wretched beings who are to inhabit them—that the *abattoirs* of the metropolis with all their reeking impurities exist in its very centre, with a slaughter-house for sheep in the cellar of every butcher in its numberless streets—that the church-yards, although loaded to the very surface, and infecting whole localities by their noisome stench during the prevalence of hot and close weather, still continue to receive the dead*—that volumes of the densest smoke, which might be profitably, and which ought to be, profitably or not, consumed, is vomited forth from all the great factories and breweries, and from the steam-boats on the river, in such profusion, that the Sunday, when a part of these nuisances is abated, is recognizable by the superior clearness and purity of the atmosphere—that our streets in snowing or wet weather are rendered impassable by reason of the accumulated filth, defying all efforts at cleanliness, and this, although thousands of poor creatures would be glad to earn an honest penny by its removal—that the sewers in the localities where they do exist are frequently choked up from their faulty construction, and become in consequence disgusting nuisances—that it is but the other day we have begun legislating for children of tender years, and woman herself is condemned to excessive or brutalizing employment, in our factories or coal mines. We might swell this list of faults of omission and commission to many pages, but refrain, merely protesting, that while such things are, we should be a little more chary in bestowing upon ourselves the epithets of a practical and common sense, a clean, and a humane people. We may, however, justly term ourselves an impartial people in the distribution of these nuisances, for if the vicinities of St. Giles and of Whitechapel reek with impurities and fever-generating nastiness, the country residence of our Queen is surrounded by similar agreeables; and if numbers of our population are swamped in superfluous moisture and poisoned by our dingy atmosphere, her London palace is built in a marsh, opposite a stagnant pond rich in malaria, and is ever and anon obscured from sight by the unconsumed smoke of the great Pimlico brewery.

The only means of taking cognizance of nuisances (besides indictment) annoyances, &c., and that to a limited extent, seems to be by, the Leet Jury—a body of perambulating honest citizens, who when first established might have been efficient, but who, in the present day, like the Coroner's inquest, and other of our tribunals, form but a clumsy and imperfect piece of mechanism for attaining the ends in view. Tailors, grocers, butchers, &c., taken pell-mell from the vicinity, are ill-qualified to perform functions requiring the skill of the surveyor, the science of the physiologist, the experience of the physician, and the patience of the statistical enquirer. Neither private individuals nor this body can act against a nuisance without all the annoyance, uncertainty, and expense of a prosecution.

Mr. Chadwick's suggestions are deserving of attention. He maintains that the road-cleansing and sewerage, including house-drainage, would be rendered less expensive and far more efficient, by consolidating the ope-

---

* The immediate vicinity of one of the worst of these disgusting nuisances has been chosen for King's College Hospital.

rations of the various districts, and submitting them to the superintendence and management of efficient *responsible* and scientific officers—rendering geological strata and not topographical divisions the basis of operation. He does not consider that permanent *boards of health*, composed of medical men, which have frequently been suggested, would prove sufficiently practical in their proceedings, and that the first improvements lie more in the province of the surveyor and engineer than the physician. In lieu of these, he proposes it should be one of the additional duties of the medical and relieving officers of the various Poor-Law Unions, to investigate the condition, and to report on the defects of the sanitary condition of the habitations of the poor which they visit; and that, upon their representations, the local executive powers should have power to forthwith act for the remedying the same. A more efficient system of inspection could not be devised, as the duties of these officers bring them necessarily into all the worst abodes and refuges of misery in their various localities. For these duties, he admits, they should be *remunerated*, as being extra to their proper functions, and unless they were so, we hope that the ruling powers would not have the effrontery to demand these arduous services at the hands of those who are already overworked in proportion to their pay. The disinterestedness and generosity of medical men have been carried so far, that in all inquiries, reports, certificates, &c. it is at once, as a matter of course, concluded they will give their services gratuitously. Even in the work we are now reviewing, we find the following note to the directions to the Assistant Commissioners, for obtaining laborious reports from leading medical men, involving great sacrifice of time and responsibility.

"3. In large towns, to request some physician, or general practitioner of eminent reputation, to prepare a report (founded upon data obtained from the various dispensaries, infirmaries, fever hospitals, and other similar establishments) respecting the circumstances promoting the prevalence of contagious and infectious disease in such large towns.

"The Commissioners will be glad to receive from you the name of any physician or general practitioner of sufficient eminence* to obtain the requisite data.

"*Note.* The Commissioners have no money to remunerate physicians; and those named should be distinctly informed that the service will be purely honorary."—*Circular Letter*, xii.

It is much to be regretted indeed that the remuneration of the poor-law medical officers is not such as to enable them to attend exclusively to their public duties, without reference to private practice, in which case, the various researches in medical statistics, and inquiries similar to the present instituted by the Commissioners, might properly be assigned to them.

Mr. Chadwick thinks a system of *inspection* would be acceptable to the profession and serviceable to the public.

"Medical officers, as private practitioners, are often dependent for their important private practice, and even for their office, on persons whom its strict performance might subject to expense, or place in the position of defendants.

* The reader will observe that when the Commissioners get them for nothing, they are laudably anxious that these services should be of the first quality. It is quite another thing when they pay for them, as their contract system witnesses.

Under such circumstances it is not unfrequent to hear the expression of a wish from these officers, that some person unconnected with the district may be sent to examine the afflicted place, and institute the proper proceedings. The working of the provisions of the Factory Act, for the limitation of the hours of labour of children, has been much impeded by the difficulty of obtaining correct certificates of age and bodily strength from private medical practitioners. On this topic a large mass of evidence might be adduced, shewing the unreasonableness of expecting private practitioners to compromise their own interests by conflicts for the public protection with persons on whom they are dependent." 350.

After an allusion to the valuable services rendered by Drs. Arnott, Smith, and Kay, Mr. Chadwick continues—

"But the results of such occasional visits appear to prove the necessity and economy of an increase of the permanent local medical service, and to establish a case for the appointment of a *superior medical man for a wider district* than an ordinary medical officer, for the special aid and supervision of the established medical relief. It will be frequently found that there is the like need of immediate local inspection of the medical treatment of the destitute that there is of a grade of inspecting surgeons for the military hospitals. It cannot be otherwise than that, amidst a numerous body of men, there must be much error and neglect in the treatment of the destitute, in the absence of immediate securities against them. The most able of the guardians would confess, that if they are not entirely incompetent to supervise medical service, they are at the best but imperfectly qualified for such a task, and the medical officers would act with more satisfaction to themselves from the supervision of officers from whom they might derive aid and confidence.

"But, besides the medical treatment of the inmates of the workhouses and prisons, there are other cases within most districts which need the preventive service of a superior medical officer for the protection of the public health. 1st. In the cases where the poorer classes are assembled in such numbers as to make the assemblages *quasi* public, and afford facilities for medical inspection, as in schools. 2nd. Also in places of work and workmen's lodgings. The occasional visitation of a district officer, for the prevention of disease, would lead to the maintenance of due ventilation, and to the protection of the work-people on such points as are already specified as injurious to the health, and that arise simply from ignorance, and are not essential to the processes. An examination of such places, if only quarterly, would lead to the most beneficial results.

"So far as I have observed the working of the Factory Act, it appears to me that the duties now performed by the sub-inspectors of factories, might be more advantageously performed by superior medical officers of the rank of army surgeons, who are independent of private practice.

"The superior economy of preventive sources by such inspection will scarcely need elucidation. * * * * * * "One of the most important services of a superior medical officer of a district would be to ensure the entries of the causes of death with the care proportionated to the important uses to be derived from them (*e. g.* as a means of guiding the direction of preventive services.) The public should be taught to regard correct registration, as being frequently of as much importance for the protection of the survivors, as a *post-mortem* examination is often found to be." 352.

Mr. Chadwick further urges the advantage of consolidating this medical service and extending it over a sufficient field of operation.

"Indeed, the money now spent in comparatively fragmentious and unsystemised local medical service for the public, would, if combined as it might be without disturbance on the occurrence of vacancies, afford advantages at each step of the combination. We have in the same towns public medical officers as inspectors of prisons, inspectors of lunatic asylums, medical officers of the new unions, medical inspectors of recruits, medical service for the granting certificates under the Factory Act, &c. These and other services are divided in such portions as only to afford remuneration in such sums as £40, £50, or £80, each; and many smaller and few larger amounts.

"Whatever may be yet required for placing the union medical officers on a completely satisfactory footing, the combination of the services of several parish doctors in the service of fewer union medical officers, will be found to be advances in a beneficial direction. The multiplication or the maintenance of such fragmentitious professional services is injurious to the public and to the profession. It is injurious to the profession by multiplying poor, ill-paid, and ill-conditioned professional men. Although each may be highly paid in comparison with the service rendered, the portions of the service do not suffice for the maintenance of an officer without the aid of private practice; (nor do the new poor-law unions, with their additional laborious and onerous duties. *Rev.*) they only suffice, therefore, to sustain needy competitors for practice in narrow fields. Out of such competition the public derive no improvement in medical science, for science comes out of wide opportunities of knowledge and study, which are inconsistent with the study to make interests, and the hunt for business in poor neighbourhoods." 354.

We have no space to enter into any criticism of the above opinions, but content ourselves with laying them thus fully before our readers.

To conclude: we have only to repeat what we stated at the commencement of this article, our opinions, and in which, after the analysis we have presented, we doubt not our readers agree, that the work is a most valuable one; and to express our regret that our limits prevent us availing ourselves of the various extracts from interesting evidence, reports, and enquiries with which it abounds. It is also illustrated with several plates. As we suppose its price is reasonable, (it ought to be sold at cost price, and probably is,) we hope it will be found in many of our readers' libraries, ranged with the works of Quetelet, Porter, Hawkins, Villermé, Parent du Chatelet, and other valuable statistical and economical writers. Facts and figures, thus accumulated by able hands, distorted, as they too often now are, by hasty, prejudiced, and superficial enquirers, will one day form, wielded by genius, the key for the solution of the sublimest problem ever proposed by human creature—the greatest happiness of the greatest numbers.

